# Microplastic Presence in the Digestive Tract of Pearly Razorfish *Xyrichtys novacula* Causes Oxidative Stress in Liver Tissue

**DOI:** 10.3390/toxics11040365

**Published:** 2023-04-11

**Authors:** Amanda Cohen-Sánchez, Antònia Solomando, Samuel Pinya, Silvia Tejada, José María Valencia, Antonio Box, Antoni Sureda

**Affiliations:** 1 Research Group in Community Nutrition and Oxidative Stress (NUCOX), University of Balearic Islands, 07122 Palma de Mallorca, Spain; 2 Interdisciplinary Ecology Group, Department of Biology, University of the Balearic Islands, 07122 Palma de Mallorca, Spain; 3 Health Research Institute of Balearic Islands (IdISBa), 07120 Palma de Mallorca, Spain; 4 Laboratory of Neurophysiology, University of the Balearic Islands, 07122 Palma de Mallorca, Spain; 5 CIBER Fisiopatología de la Obesidad y Nutrición (CIBEROBN), Instituto de Salud Carlos III (ISCIII), 28029 Madrid, Spain; 6 LIMIA-Laboratori d’Investigacions Marines i Aqüicultura, 07157 Port d’Andratx, Spain; 7 Department of Agricultura, Ramaderia, Pesca, Caça i Cooperació Municipal, Consell Insular d’Eivissa, 07800 Eivissa, Spain

**Keywords:** marine litter, antioxidant, Balearic Islands, razorfish, glutathione, plastics

## Abstract

Plastic pollution in the oceans is a growing problem, with negative effects on exposed species and ecosystems. *Xyrichtys novacula* L. is a very important fish species both culturally and economically in the Balearic Islands. The aim of the present study was to detect and categorise the presence of microplastics (MPs) in the digestive tract of *X. novacula*, as well as the existence of oxidative stress in the liver. For this purpose, the fish were categorised into two groups based on the number of MPs observed in the digestive tracts: a group with no or low presence of MPs (0–3 items) and a group with a higher presence of MPs (4–28 items). MPs were found in 89% of the specimens analysed, with a dominance of fibre type and blue colour. Regarding the type of polymer, polycarbonate was the most abundant, followed by polypropylene and polyethylene. For the group with a greater presence of MPs, the activities of the antioxidant enzymes glutathione peroxidase and glutathione reductase, as well as the phase II detoxification enzyme glutathione s-transferase, were higher than the activities observed in fish with little to no presence of MPs. The activities of catalase and superoxide dismutase and the levels of malondialdehyde did not show significant differences between both groups. In conclusion, these results demonstrate the presence of MPs in the digestive tract of *X. novacula* and the existence of an antioxidant and detoxification response, mainly based on the glutathione-based enzymes.

## 1. Introduction

One of the most serious problems worldwide is the progressive accumulation of potentially toxic waste in all ecosystems. Among them, plastics account for an important part of these pollutants that end up accumulating as litter in the marine environment [1,2]. Plastic is made up of synthetic or semi-synthetic organic polymers formed by repeating chemical structural units that give them great versatility, durability, and resistance to corrosion. These properties have caused the production of plastics to have increased exponentially in recent years [3]. Due to this extended plastic production and generalized poor waste management, a large proportion of these plastic items end up in the marine environment. Plastic may enter the marine environment in all shapes and sizes, from microplastics (MPs) from cosmetics or clothing to macroplastics [4]. Due to their high resistance, plastics do not generally biodegrade. Instead, they break down into microplastics [5]. In terms of plastic ingestion, MPs are the largest potential threat and their impact has been widely studied [6,7,8]. Due to their small size and immense ubiquity [9], they are easily ingested by most of the species in the food chain and can therefore be ingested through the predation of larger animals, and can even bioaccumulate [4,10]. The effects of plastic ingestion in marine wildlife are still widely unknown [11]. In addition to intestinal blockage and potential choking hazards [12,13,14], plastics are made with certain additives that give them their properties, such as colour, durability, or flexibility [15]. These additives are inherently toxic [16] and therefore can cause negative effects on the exposed species. In addition, plastics are also known to accumulate bacteria and chemical pollutants on their surface [17,18]. Both the additives present in plastic and the bacteria and pollutants that adhere onto the surface can, when ingested, leach into the blood stream and have negative impacts on the health of the individuals [11,19].

The presence of pollutants can induce stress in organisms, leading to the excessive production of reactive oxygen species (ROS), which have the potential to cause oxidative damage [20,21]. The deleterious effects of ROS include the alteration of the cellular redox balance, the peroxidation of membrane lipids, mainly polyunsaturated fatty acids, the inactivation of transporter enzymes and proteins, and DNA damage [22]. To protect themselves against this oxidative damage, organisms develop antioxidant defence mechanisms, which neutralise ROS and limit cell damage [23,24]. These defence mechanisms include antioxidant enzymes such as catalase (CAT), superoxide dismutase (SOD), and glutathione peroxidase (GPx), which is dependent on glutathione reductase (GRd) to regenerate glutathione [25]. Other enzymes include glutathione S-transferase (GST), which is a phase II enzyme in the detoxification process [26,27]. Plastic polymers and associated substances, including additives or adsorbed substances, can be intrinsically harmful to the exposed organisms [28]. In this sense, oxidative stress and detoxifying biomarkers can be used to assess the potential effects of plastic ingestion on marine wildlife. Solomando et al. (2020, 2022) [20,29] studied the oxidative stress response to MP ingestion in *Sparus aurata* gut and *Seriola durmilierii* liver, respectively, whilst Hoyo-Alvarez et al. (2022) [30] studied oxidative stress in seabream brains, reporting the induction of the protective system. Similarly, oxidative stress was also evident in *Eriocheir sinensis* liver tissue when exposed to MPs [31]. Qiao et al. (2019) reported intestinal inflammation, oxidative stress, and disorders of the metabolome and microbiome in zebrafish gut due to the presence of MPs [32], whilst for *Cyprinus carpio* var. larvae, MP ingestion not only caused oxidative stress, but also growth inhibition [33]. Similar studies have also proven the potential damaging effects of MPs on many aspects of the organisms’ life, endangering growth, health, and reproduction, with effects on biomarkers in different tissues when in the presence of MPs [34,35]. This evidences the possible dangers that this type of pollution can cause to marine wildlife and the normal functioning of their life cycle and population dynamics.

*Xyrichtys novacula* (Linnaeus, 1758), or pearly razorfish, is a small wrasse that lives in coastal areas of the Atlantic Ocean and the Mediterranean Sea [36]. It is very popular in the Balearic Islands, Spain, where it is one of the main targets of recreational fishing [37] and is very popular in the cuisine [38]. This fish lives close to the seafloor [39,40] where it buries for protection [41]. It has been found to feed both in this sediment and in the water column [36,38,42]. It has a very small home range and is very territorial [41], and therefore can be an interesting biomarker for plastic presence both in the water column and in sediment. Its high popularity as a target species also makes it a valuable research subject from a socioeconomic standpoint.

Although MP ingestion has become a popular field of study [6,7,8,43,44,45,46], the potential adverse effects that these plastics can have on marine wildlife are only starting to become understood. In addition, most studies have been carried out under controlled laboratory conditions and not in the natural environment. In this sense, the aim of the present study was to evaluate the presence of MPs in wild *X. novacula* sampled in the islands of Ibiza and Formentera (Balearic Islands, Spain), as well as the capability of MPs to induce oxidative stress on these fish through the study of biomarkers in their liver tissues.

## 2. Materials and Methods

### 2.1. Experimental Procedure

**Figure 1:**
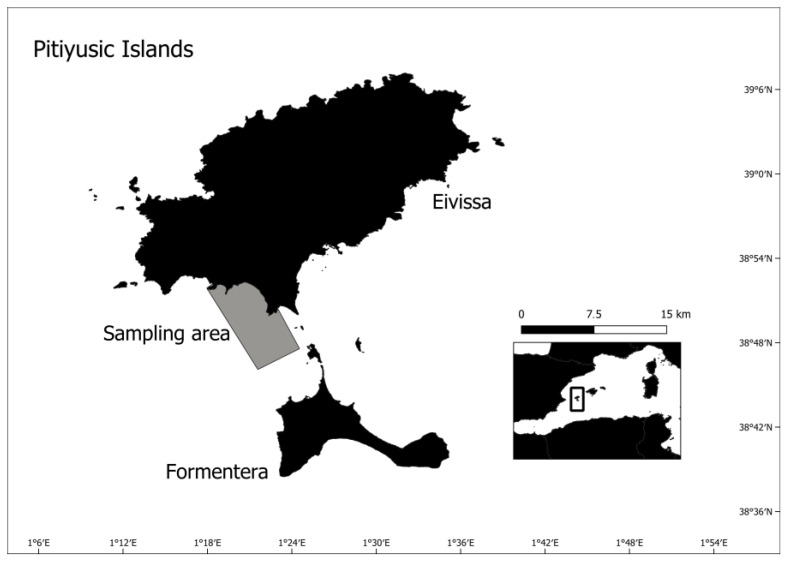
Location of Eivissa Island (Balearic Islands, Spain, Western Mediterranean) with the sampling area in grey colour.

During October 2020, 36 razorfish were fished in the southeast region of the island of Ibiza with a hook using worms as bait (Figure 1). The sampling area is far from the main population centres in order to reduce the influence of other possible contaminants. The fish were anesthetised with tricaine methanesulfonate (MS-222) (1 g/10 L water) to minimise stress. The individuals were measured (total length: ±0.1 cm and total weight: ±0.1 g), sexed, and the gastrointestinal tract and the liver were dissected. The gastrointestinal tract was frozen at −20 °C until further analysis in the laboratory, whereas the liver was immediately introduced into liquid nitrogen and kept at −80 °C in the laboratory until biochemical analyses. The experimental procedure followed the EU Directive 2010/63/EU for animal experiments and was revised and approved by the Ethics Committee for Animal Experimentation of the University of the Balearic Islands (Ref. 020/06/AEXP).

### 2.2. Microplastic Analysis

The gastrointestinal tracts were digested in Erlenmeyer flasks with 20 mL of 10% KOH solution per gram of digestive tissue following a previously described procedure [47]. Briefly, after complete digestion of the tissue, the samples were filtered with a Büchner funnel, using filtering paper of 47 mm in diameter and a 10 µm pore size (Albet LabScience, Barcelona, Spain), and the filters were then dried at 60 °C. Control blanks were also carried out simultaneously under the same conditions as the samples to account for possible environmental contamination. MPs captured in the filters were visually identified and photographed through a Leica EZ4 stereomicroscope with an HD camera (optical magnification up to 11.5×). During the handling process, measures to avoid microplastic contamination were adopted, such as working under a laminar flow hood, rinsing and cleaning material and surfaces using filtered ethanol 70% and MilliQ water, and wearing lab coats made of 100% cotton. The MP particles were categorised by colour, size, and type (fibre or fragment).

The polymer composition of the MPs (N = 46 items) was analysed using micro-attenuated total reflection micro-Fourier-transform infrared spectroscopy (μ-ATR-FTIR) (Bruker, OPTICS, Germany). Due to the small size of the MPs, analysis was carried out with the Hyperion ATR microscope. FTIR absorption spectra were recorded as an average of 250 scans in the mid-infrared range of 400–4000 cm^−1^. The obtained spectrum was compared with commercial and in-house spectral databases, and a minimum of 700 hit quality index was necessary to accept a confirmed polymer [48].

### 2.3. Biochemical Analysis

Liver sections of *X. novacula* were homogenised in ten volumes (*w*/*v*) of Tris–HCl buffer 100 mM, pH 7.5 using a dispersing system (Ultra-Turrax^®^ Disperser, IKA, Staufen, Germany). The samples were then centrifuged at 9000× *g* for 10 min at 4 °C, and supernatants were recovered and used for biochemical analyses.

The activities of the antioxidant enzymes CAT, SOD, GPx, and GRd, and the detoxification enzyme, GST, were monitored using a spectrophotometer (Shimadzu UV-2100, Kyoto, Japan) at 25 °C. CAT activity (mK/mg prot) was determined following the method of Aebi based on the decomposition of H_2_O_2_ at 240 nm [49]. SOD activity (pKat/mg prot) was determined according to previously described method at 550 nm, using cytochrome as an indicator [50]. GPx activity was determined at 340 nm using H_2_O_2_ and reduced glutathione (GSH) as substrates [51]. GRd activity was monitored at 340 nm with oxidised glutathione (GSSG) as a substrate [52] method. GST activity (nKat/mg prot) was determined at 340 nm using GSH and 1-chloro-2,4-dinitrobenzene (CDNB) as substrates [53]. MDA concentration (nM/mg protein) was quantified using a colorimetric assay kit (Merck, Madrid, Spain), following the manufacturer’s instructions.

All results were normalised by the protein content of the samples (Bio-Rad^®^ Protein Assay, Alcobendas, Spain) using bovine serum albumin as a standard.

### 2.4. Statistical Analysis

The effects of MP ingestion on oxidative stress biomarkers were evaluated using a statistical analysis package (SPSS 27.0 for Windows^®^) (IBM^®^ SPSS Inc., Chicago, IL, USA). For the statistical analysis, the samples were categorised into two groups according to the number of MPs observed in the gastrointestinal tract: low MPs (N = 18) and high MPs (N = 18). The normality of the data was confirmed by the Shapiro–Wilk test and homogeneity of variance by the Levene’s test. Statistical differences between the groups were carried out with a Student’s *t*-test for unpaired data. The correlations between the presence of MPs and stress biomarkers were calculated using the bivariate correlation Pearson test. The results are presented as mean ± standard error of the mean (SEM) and *p* < 0.05 was considered statistically significant.

## 3. Results

### 3.1. Biometric Parameters

A total of 36 fish were included in the study, with an average size of 15.6 ± 0.5 cm (15.7 ± 0.8 low MPs, 15.5 ± 0.7 high MPs) and weight of 55.5 ± 5.7 g (55.8 ± 8.9 low MPs, 55.1 ± 7.4 high MPs). Moreover, 60% of the caught fish were females. The fish were caught at a depth of approximately 15–20 m with water at 19 °C and 36 psu.

### 3.2. Microplastics in the Gastrointestinal Tract

**Figure 2:**
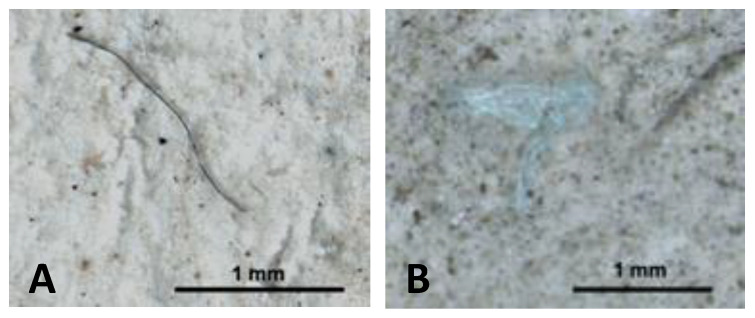
Representative images of a microplastic fibre (**A**) and fragment (**B**) found in the gastrointestinal tract of *Xyrichtys novacula*. Scale bar represents 1 mm.

MPs were reported in 32 of the 36 (89%) the gastrointestinal tract of fish analysed with a total of 171 plastic items, while in the remaining 4, there was no evidence of MPs (11%), and in 7 of the fish, only one plastic element was found (19%). Some representative images of the MPs found in the gastrointestinal tracts of *X. novacula* are presented in Figure 2. Collectively, the fish presented an average of 4.75 ± 0.90 items/individual in their gastrointestinal tract.

To assess whether the increased presence of MPs is related to variations in oxidative stress markers, the fish were divided into two groups according to the number of MPs. Thus, the group with low MP content presented 1.28 ± 0.23 items/individual (ranging between 0 and 3 items per individual) and the group with high MP content exhibited 8.22 ± 1.36 items/individual (ranging between 4 and 28 items per individual).

When analysing the characteristics of the MPs found, 81.7% corresponded to a fibre type, while the remaining 18.7% were fragments. Regarding the colours, the most common MP colour in the samples was blue (50.9%, 87 items), followed by black (25.1%, 43 items), red (7.6%, 14 items), and grey (5.3%, 9 items).

**Figure 3:**
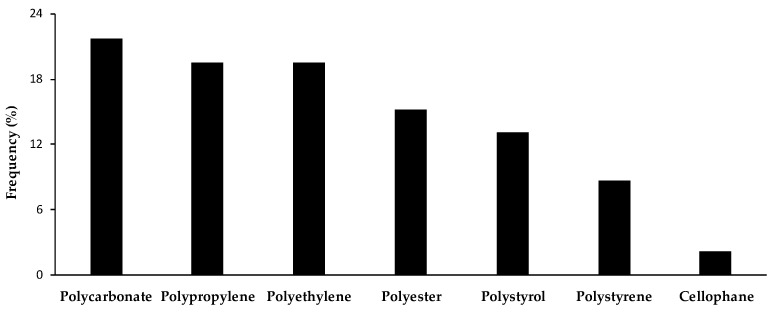
Proportion of plastic polymer type (%) found in *Xyrichtys novacula* gastrointestinal tracts in Eivissa Island (Balearic Islands, Spain).

Chemical polymer identification was carried out in 46 items using μ-ATR-FTIR (Figure 3). The most common polymers were polycarbonate (21.7%), followed by polypropylene (19.6%) and polyethylene (19.6%). The remaining polymers were polyester (15.2%), polystyrol (13.0%), polystyrene (8.7%), and cellophane (2.2%).

### 3.3. Biomarker Analysis

**Figure 4:**
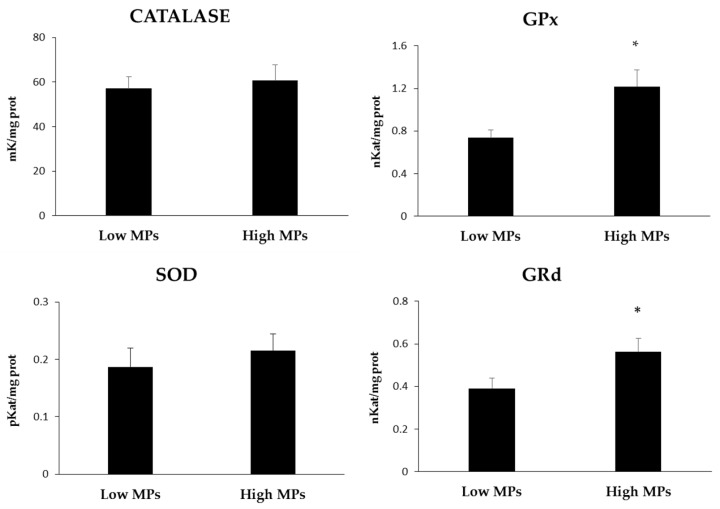
Oxidative stress biomarkers determined in the liver tissue homogenates of *Xyrichtys novacula* sampled in Eivissa (Balearic Islands, Spain). Superoxide dismutase (SOD); glutathione peroxidase (GPx); glutathione reductase (GRd). * indicates significant differences (*p* < 0.05) between groups.

The activity of the antioxidant enzymes is shown in Figure 4. All of the activities tended to be higher in the group with the highest presence of MPs, although the differences were only statistically significant for GPx and GRd (*p* < 0.05) and not for catalase and SOD.

**Figure 5:**
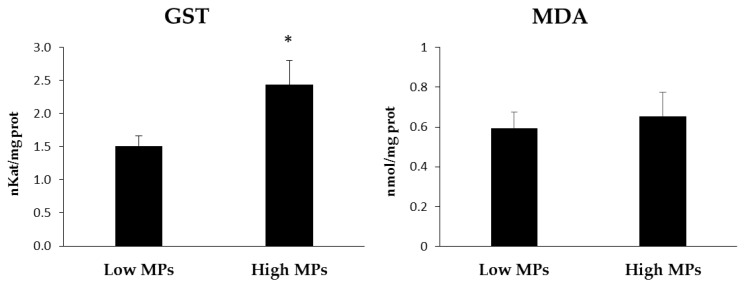
GST (glutathione *S*-transferase) activity and MDA (malondialdehyde) levels determined in the liver tissue homogenates of *Xyrichtys novacula* sampled in Eivissa (Balearic Islands, Spain). * indicates significant differences (*p* < 0.05) between groups.

GST activity also showed significantly higher values in the fish with higher MP content (*p* < 0.05) (Figure 5). For MDA, as an indicator of lipid peroxidation, although the group with more MPs had higher values, these did not reach significance. No significant differences were found for either MP quantity or biomarker variations between male and female fish.

The correlation analysis revealed the existence of a positive association between high levels of MPs and the activities of the GST (r = 0.738; *p* < 0.001), GPx (r = 0.385; *p* < 0.025), and GRd (r = 0.400; *p* < 0.017). The remaining biomarkers did not present correlations in relation to the MPs.

## 4. Discussion

The ubiquity and small size of MPs make them easily ingestible by many taxa, with potentially negative physical, physiological, and toxicological impacts [9,12,54]. The presence of MPs in the digestive tract can cause intestinal obstruction, inflammation, reduce appetite, and impair nutrient absorption [55]. In addition, smaller MPs, as well as adsorbed substances, can cross endothelial cells, enter the circulatory system, and move to other organs such as the liver, inducing toxic effects on physiological functions [56]. The present results evidenced the presence of MPs in almost 90% of the sampled individuals of *X. novacula*, suggesting that this species is prone to MP ingestion. Such a high percentage of MP-polluted individuals has been reported in several papers: 100% of the sampled deep-sea fish (N = 35, 11 species) from south China [46] and 77% of *Engraulis japonicus* (Temminck and Schlegel 1846) (N = 64) in Tokyo Bay [44]. In the Mediterranean Sea, high values, close to 100%, have been observed in *Scomber scombrus* and *Trachurus trachurus* in the south Iberian coast [57] and in *Seriola dumerili* caught around Eivissa and Formentera [29], and approximately 70% in *Boops boops* (Linnaeus 1758) (N = 337) sampled in the coastal waters of Mallorca [58]. Moreover, the presence of MPs in a benthic fish that lives on sandy and muddy bottoms similar to razorfish, such as *Solea solea* (Linnaeus, 1758) from the northern and central Adriatic Sea, reached 95% of the specimens analysed [59]. Other studies, however, have reported lower values for the percentage of fish presenting with MPs, such as 19.8% of commercial fish (N = 263, 26 different species) from Portuguese waters [43], 27.3% for *Mullus surmuletus* (Linnaeus 1758) (N = 417) captured around the coasts of Mallorca [24], and 58% of sampled species in Turkish waters (N = 1337) [60]. *X. novacula* feeds both from the water column and directly from the sediment, feeding mainly on benthic invertebrates such as molluscs, shrimps, crabs, and polychaetes [36,61], which makes it vulnerable to ingestion from both environments. In addition, it is a species that lives in coastal areas, between 5 and 50 m deep, on sandy bottoms and in *Cymodocea nodosa* seagrass meadows, generally with high anthropic pressure associated with tourism, which can favour its exposure to MPs, mostly of land-source origin [1].

The colours and types of plastics (fibre or fragment) are consistent with previous scientific literature [24,58,60,62]. Plastics are generally described as blue and black, which might be due to the main colours from the source (packaging, clothing, etc.), or mistaken for prey since they resemble blue copepod species, for example [57,63]. As for fibres, they are found in significant quantities both in the water column and in sediment [64], as well as in other species in various studies [58,62]. The main sources of fibres have been recognized to be washing machines, but also fragments from fishing nets and the textile industry [65,66].

MPs and associated components, such as plasticisers, additives, and other adsorbed pollutants, make them dangerous as they could potentially disrupt the immune and antioxidant systems [67]. This occurs when these alien components affect the cells and induce an excess and accumulation of ROS, creating a pro-oxidative state [68]. ROS are highly reactive and can interact with biomolecules, altering their structure and function and establishing a situation of oxidative stress [69]. To protect themselves, organisms have an elaborate antioxidant and detoxification system to deactivate and eliminate pollutants and to maintain ROS at physiological levels. The present study highlights the potential of MPs to induce a physiological response in *X. novacula*, since higher values of antioxidant enzymes and GST have been observed for fish with a higher number of MPs in their digestive tracts. Specifically, a greater activity of GPx and GRd, enzymes that participate in the glutathione redox cycle with a key role in the reduction of intracellular hydroperoxides and H_2_O_2_, has been observed [25]. In this sense, the correlation analysis showed a direct relationship between the number of MPs found in the gastrointestinal tract of *X. novacula* and the activities of GPx and GRd, and especially of GST. GPx catalyses the reduction of H_2_O_2_ and other peroxide radicals, preventing lipid peroxidation and avoiding cell membrane damage, whereas GRd converts the oxidised form of glutathione (GSSG) to GSH [70]. In this sense, the presence of MPs can induce oxidative stress with an increase in the production of ROS that translates into an activation of antioxidant mechanisms, especially those related to the GSH cycle. In a study carried out in the liver of juvenile large yellow croaker *Larimichthys crocea*, GPx was observed to be the first enzyme to significantly increase in the presence of nanoplastics, whereas CAT, SOD, and MDA only increased with the highest MP concentrations [71]. These results match those observed in this study, since despite all of the activities being higher in the group with a greater abundance of MPs, the activities of SOD and CAT were not significantly higher, which suggests that the presence of MPs was not important enough. These results make sense, since in most studies carried out under controlled conditions, the exposure to MPs is much higher than in the natural environment. Similarly, GPx was also reported to increase in *Cyprinus carpio* liver when exposed to a plasticiser (di-n-butyl phthalate) [72], as well as in *Carassius auratus*, exposed to similar plasticisers [73]. GRd activity has also been found to increase at low to moderate levels of plastic contamination, as seen in the gut of *Holothuria tubulosa*, where for three areas with different plastic contamination (pristine, intermediate, and contaminated), is the only biomarker tested with significantly higher values for the intermediate location [74]. Likewise, the results for *Sparus aurata* showed that both GPx and GRd increase in liver tissues when exposed to plastics [75]. In addition, the MDA levels do not show significant differences between the two groups, which would indicate that the antioxidant mechanisms prevent an increase in the oxidation of biomolecules. Similar to what occurs with antioxidants, markers of the oxidative damage associated with MPs tend to show few changes in organisms obtained from the natural environment [24,29].

GST, a metabolic isozyme involved in phase II detoxification process, favours the elimination of pollutants by conjugating GSH and transforming them into a more hydrophilic product [27]. GST activity has been found to increase when in the presence of plastic pollution, suggesting an induction of the detoxification system [24,76]. Similarly, in the present study, GST activity significantly increased in the group with high MP presence, matching the results from both Alomar et al. (2017) [20] and Solomando et al. (2020) [24], which also reported increased GST activity related to the presence of a larger amount of ingested MPs in *M. surmuletus* and *S. aurata*, respectively. Furthermore, increased GST activity was also observed in the livers of *M. surmuletus* and the brain of *B. boops* when exposed to microplastics [77], as well as in the brains of *S. aurata* subjected to an MP-enriched diet [30]. Therefore, plastic presence seems to be closely linked to GST, GRd, and GPx in the case of the studied *X. novacula* liver samples. Although further studies are needed to confirm the biomarker that is most related with MP presence, these results suggest that enzymes such as GST, GRd, and GPx could be primary indicators of plastic pollution effects on fish for small concentrations of MP ingestion. Due to the small sample size, more studies are necessary to fully understand the long-term effects of microplastic exposure fish and ecosystem health. In addition, the difficulty of finding areas without anthropic influence is a limitation, since it does not allow knowledge of the baseline activity of the measured enzymes in this fish species for comparison. In fact, significant levels of MPs have been found on the coasts of the Marine Protected Area of the Cabrera Archipelago National Park, in the south of Mallorca Island [78]. Ongoing studies with other similar species will be able to better explain the effect of MP presence in the ecosystem.

## 5. Conclusions

In conclusion, *X. novacula* is exposed to MPs, since plastic items have been found in the gastrointestinal tract of practically all of the specimens analysed. A greater presence of MPs is associated with an activation of antioxidant enzymes, especially those related to the GSH cycle, as well as the GST detoxifying enzyme. These results highlight the potentially dangerous effects of ingesting MPs on *X. novacula*, and also emphasise possible early warning enzymes for oxidative stress. However, more research is required to provide information on the short- and long-term physiological effects of the ingestion of MPs on marine species, which biomarkers are most related to the presence of MPs, and to determine whether they may affect the fish population.

## Data Availability

Researchers wishing to access the data used in this study can make a request to the corresponding author: antoni.sureda@uib.es.
